# Supplementary material for: Morphology-modified contributions of electronic transitions to the optical response of plasmonic nanoporous gold metamaterial
Source: Nat Commun. 2026 Jan 20;17:829. doi: 10.1038/s41467-026-68506-0 (PMC12824242; doi:10.1038/s41467-026-68506-0)
Supplement: Supplementary file 1 — Supplementary Information [file 41467_2026_68506_MOESM1_ESM.pdf]

Supplementary Information

Morphology-modified contributions of electronic transitions to the optical response of plasmonic nanoporous gold metamaterial

Tlek Tapani[1¶], Jonas M. Pettersson[1¶], Nils Henriksson[1¶], Carla M. Brunner[1¶], Ann Céline Zimmermann[1], Erik Zäll[2], Nils V. Hauff[3], Lakshmi Das[1], Anastasiia Sapunova[4,5], Gianluca Balestra[6,7], Massimo Cuscunà[6], Aitor De Andrés[1], Tommaso Giovannini[8], Denis Garoli[4,9], and Nicolò Maccaferri[1,10]*

1. Ultrafast Nanoscience Unit, Department of Physics, Umeå University, Umeå SE-901 87, Sweden
2. Nano for Energy Unit, Department of Physics, Umeå University, Umeå SE-901 87, Sweden
3. Umeå Centre for Electron Microscopy, Umeå University, Umeå SE-901 87, Sweden
4. Istituto Italiano di Tecnologia, Via Morego 30, Genova, 16163, Italy
5. Department of Materials Science, University of Milano-Bicocca, Piazza dell'Ateneo Nuovo 1, 20126 Milan, Italy
6. CNR NANOTEC Institute of Nanotechnology, Via per Monteroni, 73100 Lecce, Italy
7. Department of Mathematics and Physics 'Ennio de Giorgi', University of Salento, 73100 Lecce, Italy
8. Department of Physics, University of Rome Tor Vergata, Via della Ricerca Scientifica 1, I-00133 Rome, Italy
9. Dipartimento di Scienze e Metodi dell'ingegneria, Università di Modena e Reggio Emilia, Via Amendola 2, 42122, Reggio Emilia, Italy
10. Wallenberg Initiative Materials Science for Sustainability, Department of Physics, Umeå University, Umeå SE-901 87, Sweden

*nicolo.maccaferri@umu.se

¶These authors contributed equally

1. X-ray photoelectron spectroscopy core-level spectra of bulk and nanoporous gold
2. Pump and probe pulses characterization
3. Linear absorption measurements and simulations
4. Calculation of the permittivity change induced by the pump
5. Calculated transient change in permittivity
6. X-ray photoelectron spectroscopy valence-band spectra of bulk and nanoporous gold
7. Representing different systems with Bruggeman model
8. Transient transmission as a function of filling factor of nanoporous gold

Supplementary Note 1. X-ray photoelectron spectroscopy core-level spectra of bulk and nanoporous gold

We recorded Au 4*f* core-level XPS spectra of NPG, BG, and a reference Au film (grown directly inside the XPS chamber). The peaks show identical binding energies and linewidths within our experimental uncertainty (~0.1 eV)[1]. The absence of measurable chemical shifts or broadening indicates no detectable oxidation, alloying, or differential charging between BG and NPG. Thus, the phenomenology discussed in the main text is unlikely to arise from a change in the chemical state of Au; instead, it is consistent with morphology-driven effects that influence the electronic processes and the optical response of NPG. Representative images of the NPG are reported Supplementary Figure 2.

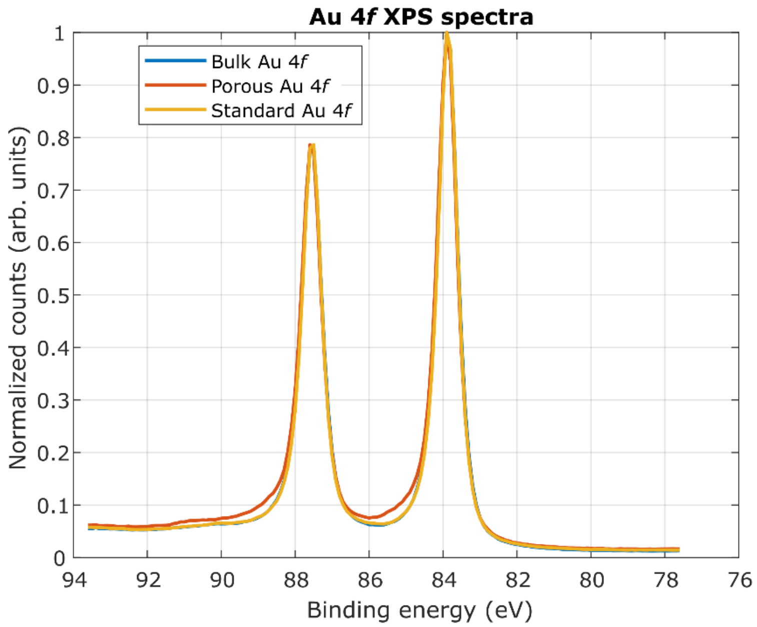

Supplementary Figure 1. **XPS spectra of samples under study.** 4*f* core-level XPS spectra of BG (blue curve), NPG (red curve) and reference Au (yellow curve) films.

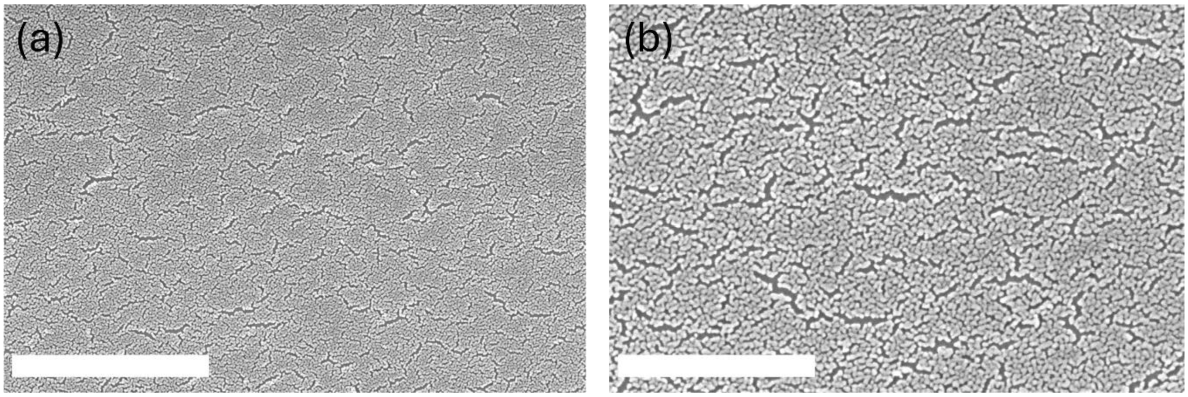

Supplementary Figure 2. **SEM images of the NPG. a** Low magnification image with a scale bar of 1 μm. **b** Higher magnification image revealing finer morphological details, scale bar 500 nm.

Supplementary Note 2. Pump and probe pulse characterization

To characterize the pulse duration and compression of our pump and probe pulses, we used a home-made second harmonic frequency resolved optical gating (SHG-FROG) setup, employing 10-µm-thick beta Barium Borate (BBO, Eksma Optics) crystals optimized for each pulse's central wavelength, and all-reflective optics. We measure the generated second harmonic with a calibrated Spectrometer (Thorlabs Compact Spectrometer CCT10). The measured and reconstructed SHG-FROG traces of the pump and probe pulse are shown in Supplementary Figure 2a and Supplementary Figure 3a, respectively. From this, we can reconstruct the spectrum and phase of the fundamental pump and probe pulses. These are shown in Supplementary Figure 2b and Supplementary Figure 3b, respectively, in solid lines, together with the corresponding spectra measured directly with the same spectrometer (dashed lines). The reconstructed pump pulse spectrum shows excellent agreement with the directly measured spectrum, while the probe pulse exhibits some discrepancies between the two spectra. The main reason is because (i) the compression range of the chirped mirrors only works in the range of 500 – 750 nm and (ii) by the limited phase matching of the BBO crystal, which could be achieved only for wavelengths between 550 nm and 780 nm. However, given that in our study we focus on the well-compressed wavelength region (550-750 nm), this deviation does not affect the validity of our measurements. In Supplementary Figure 2c and Supplementary Figure 3c we show the time traces of the pump and probe pulse, respectively. We get an FWHM time duration of 12 fs for the pump pulse and 11 fs for the probe pulse. These results entail that our time resolution can is below 15 fs, considering cross-correlation between pump and probe pulses when they temporally overlap.

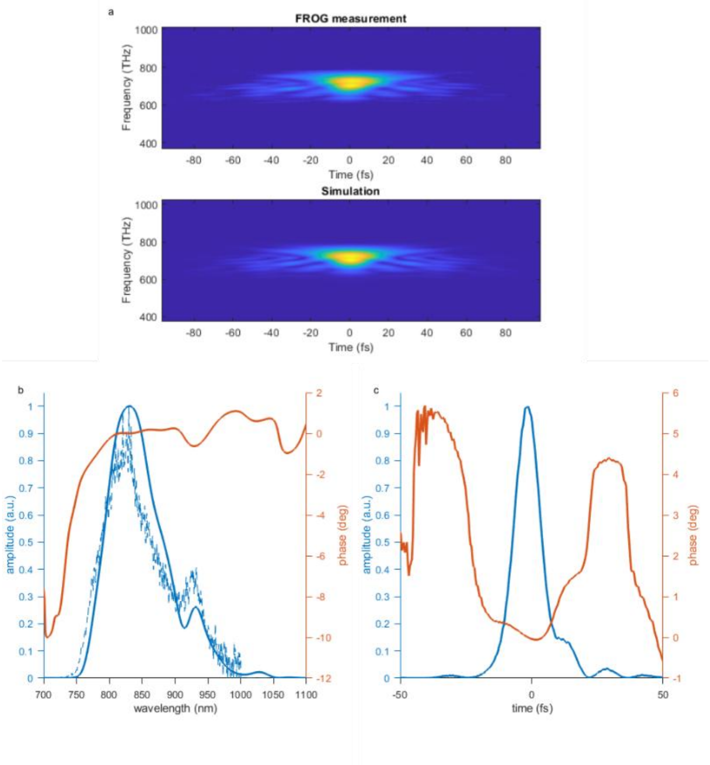

Supplementary Figure 3. **Temporal characterization of the pump pulse via a home-built SHG-FROG.** The device utilizes all-reflective optics and a 10-µm-thick BBO crystal (Eksma Optics). **a** Measured and reconstructed SHG-FROG traces. **b** Reconstructed spectrum (blue) and phase (orange) via SHG-FROG measurement (solid lines). The pump spectrum measured with a spectrometer is shown as dashed blue line. **c** Reconstructed time trace of the pump pulse via SHG-FROG measurement with an FWHM of 12 fs.

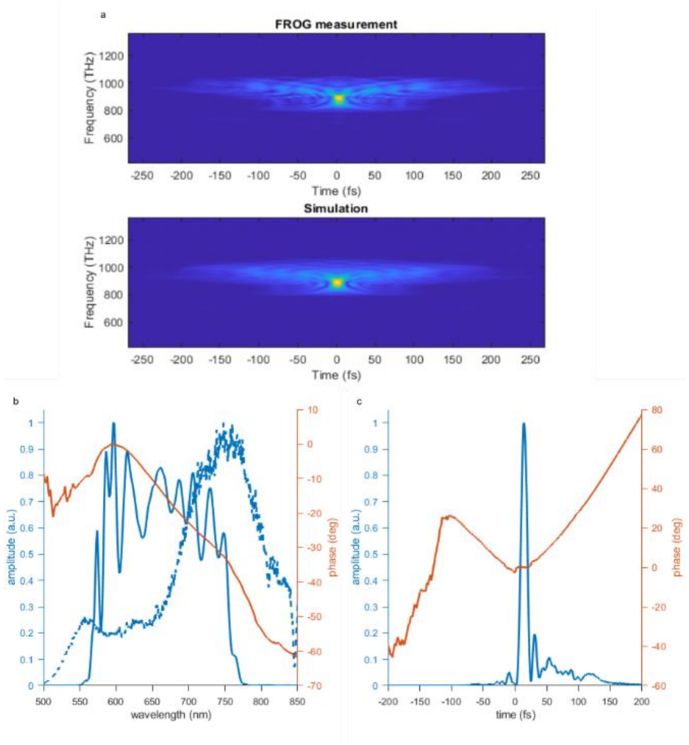

Supplementary Figure 4. **Temporal characterization of the probe pulse via home-built SHG-FROG. a** Measured and reconstructed SHG-FROG traces. **b** Reconstructed spectrum (blue) and phase (orange) via SHG-FROG measurement in solid lines. The probe spectrum measured with a spectrometer is shown as dashed blue line. Discrepancies between SHG-FROG spectrum and spectrum measured with spectrometer arise from limited phase matching spectral range and an uncompressed probe component around 750nm. **c** Reconstructed time trace of the probe pulse via SHG-FROG measurement with an FWHM of 11 fs.

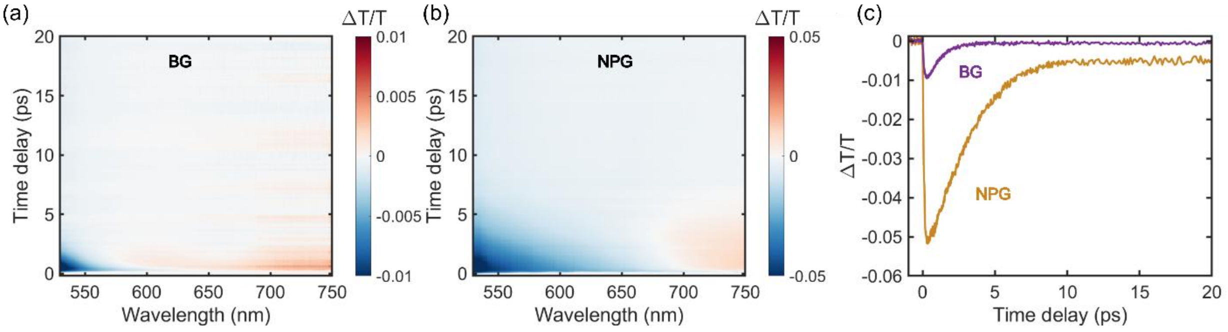

Supplementary Figure 5. **Pump probe measurements up to 20 ps time delay. a** $\Delta T/T$ of BG film as a function of time delay between pump and probe pulses and wavelength of the probe pulse. **b** $\Delta T/T$ of NPG films. **c** $\Delta T/T$ of NPG (yellow curve) and BG (purple curve) films as a function of the time delay between pump and probe pulses at 540 nm.

Supplementary Note 3. Linear absorption measurements and simulations

To measure the linear absorption of our samples, we used a spectrophotometer (model: PerkinElmerLAMBDA 1050+ UV/Vis/NIR) to measure transmittance (T) and hemispherical reflectance (R). Using conservation of energy, absorption (A) is retrieved as: $A = 1 - T - R$. Supplementary Figure 5 compares the linear optical spectra of (a) NPG and (b) BG films (continuous lines). The dashed lines represent the calculations of the optical spectra using the transfer matrix method (TMM) and the permittivity from Rakic[2]. The largest difference between these two is the reduction of reflectance and increase of transmittance in NPG film, which can be explained by the nanoporous structure. The porous surface causes higher scattering and the rise in transmittance is due to a smaller filling factor, decreasing the effective extinction coefficient of the film.

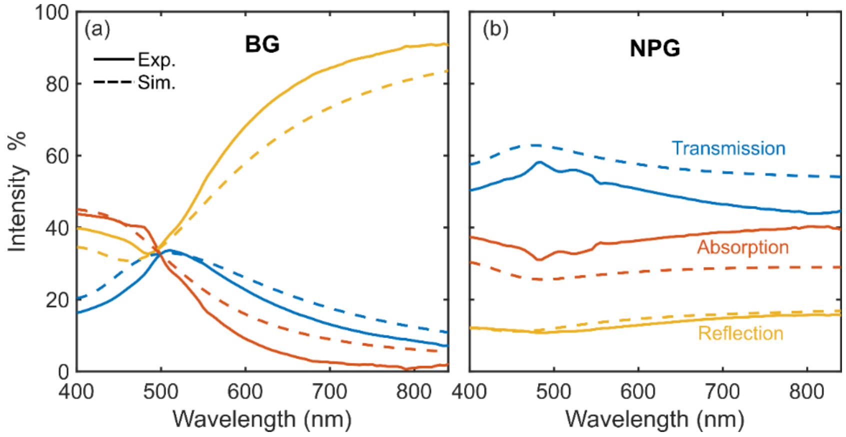

Supplementary Figure 6. **Optical response of bulk and nanoporous gold films. a** Experimental/calculated transmittance (continuous/dashed blue lines), reflectance (continuous/dashed yellow lines) and absorption (continuous/dashed red line) of BG. **b** Experimental/calculated transmittance (continuous/dashed blue lines), reflectance (continuous/dashed yellow lines) and absorption (continuous/dashed red line) of NPG.

Supplementary Note 4. Calculation of the permittivity change induced by the pump

The interband contribution to the permittivity change was calculated following previous works[3–5]. The variation of the permittivity follows a change to the joint density of states for valence to conduction band transitions,

$$\Delta J_{\mathrm{L,X}} = -\int_{E_{\mathrm{min}}}^{E_{\mathrm{max}}} D(E,\omega)\Delta f(T,E)\mathrm{d}E, \tag{1}$$

where D is the energy distribution of the joint density of states, $\Delta f(T,E) = f(T,E) - f(T_0,E)$ the change in the Fermi function f and T the temperature of the electrons. The derivations of J, D as well as the integration limits are derived in works by Rosei and co-workers[6,7]. L and X represent the corresponding symmetry points in the first Brillouin zone of Au and are the two points supporting interband transitions following excitation of visible light. The change in permittivity is then determined as

$$\Delta\varepsilon'' = \frac{8\pi^2 e^2 \hbar^4}{3m^2} \frac{\left|\mathbf{p}_{\mathrm{v,c}}^{\mathrm{L}}\right|^2}{(\hbar\omega)^2} \Delta J_{\mathrm{L}} + \frac{8\pi^2 e^2 \hbar^4}{3m^2} \frac{\left|\mathbf{p}_{\mathrm{v,c}}^{\mathrm{X}}\right|^2}{(\hbar\omega)^2} \Delta J_{\mathrm{X}}, \tag{2}$$

where m is the electron mass, $\mathbf{p}_{\mathrm{v,c}}^{\mathrm{L,X}}$ the dipole matrix element between the valence and the conduction band at the L and X points, and $\hbar\omega$ the photon energy of the probe. Here, we assume that the matrix elements are constant, and thus used them as fitting parameters following the approach described by Rosei et al.

Finally, the intraband contribution to the permittivity change was calculated by accounting for changes to the damping and the plasma frequency in the Drude model with lattice temperature. The change for the damping was determined as

$$\Delta\Gamma(T_{\mathrm{l}}) = \beta(T_{\mathrm{l}} - T_0)\Gamma_{\mathrm{e-ph}}. \tag{3}$$

Here, T_{l} is the lattice temperature, ω is the frequency of the probe, $\Gamma_{\mathrm{e-ph}} = 4.56 \times 10^{13}$ rad/s the contribution to the damping following an increase in temperature and $\beta = 3.3 \times 10^{-3}$ K^{-1}, see Refs. [4,8] and references therein. Similarly, the change to the plasma frequency, which stems from a decrease in the electron density due to thermal expansion, was calculated as

$$\Delta\omega_{\mathrm{P}}(T_{\mathrm{l}}) = -\alpha(T_{\mathrm{l}} - T_0)\omega_{\mathrm{P}}, \tag{4}$$

where $\omega_{\mathrm{P}} = 1.327 \times 10^{16}$ rad/s the plasma frequency and $\alpha = 4.5 \times 10^{-5}$ K^{-1} the thermal expansion coefficient.

The change in permittivity was determined as $\Delta\varepsilon = \Delta\varepsilon' + i\Delta\varepsilon''$, while also altering the Drude parameters for the room-temperature permittivity by adding the changes from Equations (3) - (4). $\Delta\varepsilon'$ is the real part of the interband permittivity change determined using Kramers-Kronig relations on $\Delta\varepsilon''$.

Supplementary Note 5. Calculated transient change in permittivity

By using our model based on e2TM and TMM, we tested if the observed effects in Figure 1 of the main text have a more trivial origin. By looking at the real and imaginary part of the permittivity change after pump excitation (see Supplementary Figure 6 below), we see that the imaginary part, which is proportional to the absorption, is positive in both BG and NPG cases, indicating that the observed effects are linked to probe light absorption and not to ground state bleaching.

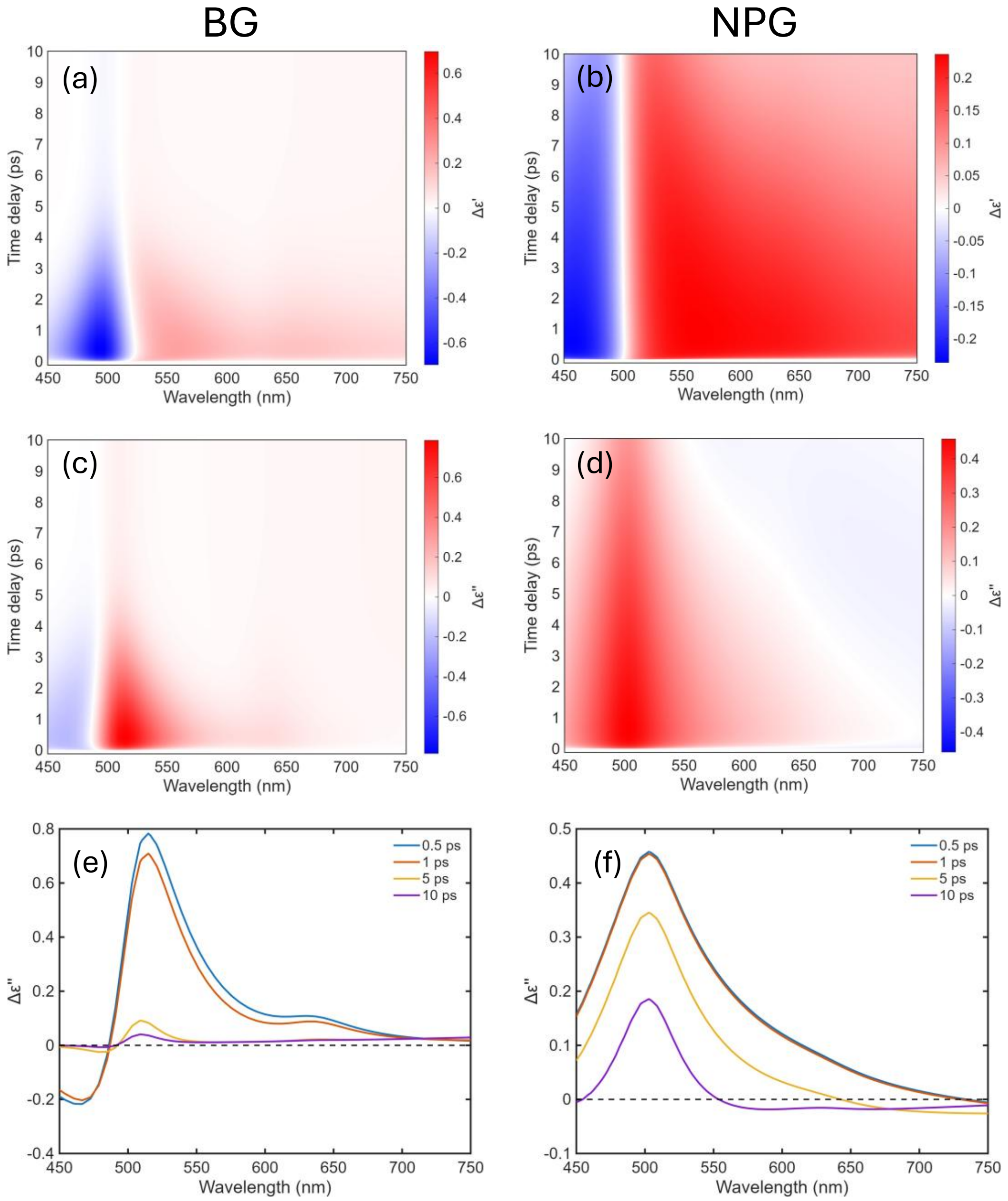

Supplementary Figure 7. **Calculated transient permittivity as a function of time delay between pump and probe pulses (vertical axis) and wavelength of the probe light pulse (horizontal axis) using the e2TM and the TMM.** **a,b** Real part for BG and NPG. **c,d** Imaginary part for BG and NPG. **e,f** Imaginary transient permittivity (proportional to the absorption) for BG and NPG at different time delays between pump and probe.

Supplementary Note 6. XPS valence-band spectra

We recorded valence-band XPS spectra for NPG, BG, and a reference Au film. The Fermi edge and Au 5*d* features coincide in position and lineshape with no discernible energy shift, within our experimental uncertainty (~0.1 eV). These data indicate no measurable change in the valence-band electronic structure between BG and NPG, consistent with the conclusion that the differences discussed in the main text arise from macroscopic effects, i.e., sample morphology (porosity) rather than changes in the electronic bands.

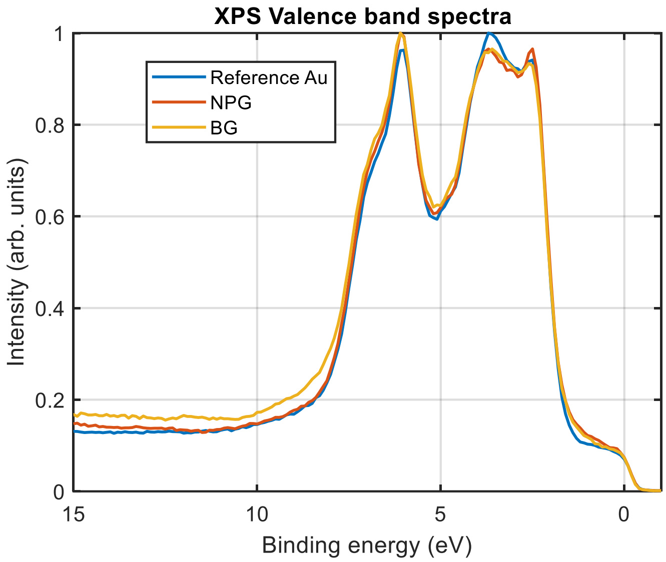

Supplementary Figure 8. **Normalized valence-band XPS spectra for BG, NPG and reference Au films.**

Supplementary Note 7. Representing different systems with Bruggeman model

By changing the depolarization factor in Equation (1) of the main manuscript, our model can represent different systems. In Supplementary Figure 8, we show what the calculated $\Delta T/T$ signal as a function of the wavelength looks like at the time delay corresponding to the maximum signal. Specifically, in Supplementary Figure 8**a** we present results for a BG film, as well as inclusions in the shape of very thin rods ($\nu_{x,y} = 1/2, \nu_z = 0$) as well as disks ($\nu_{x,y} = 0, \nu_z = 1$)[9]. While there are many inclusions that can be represented, the system must be a mixture representable by the Bruggeman effective medium approximation.

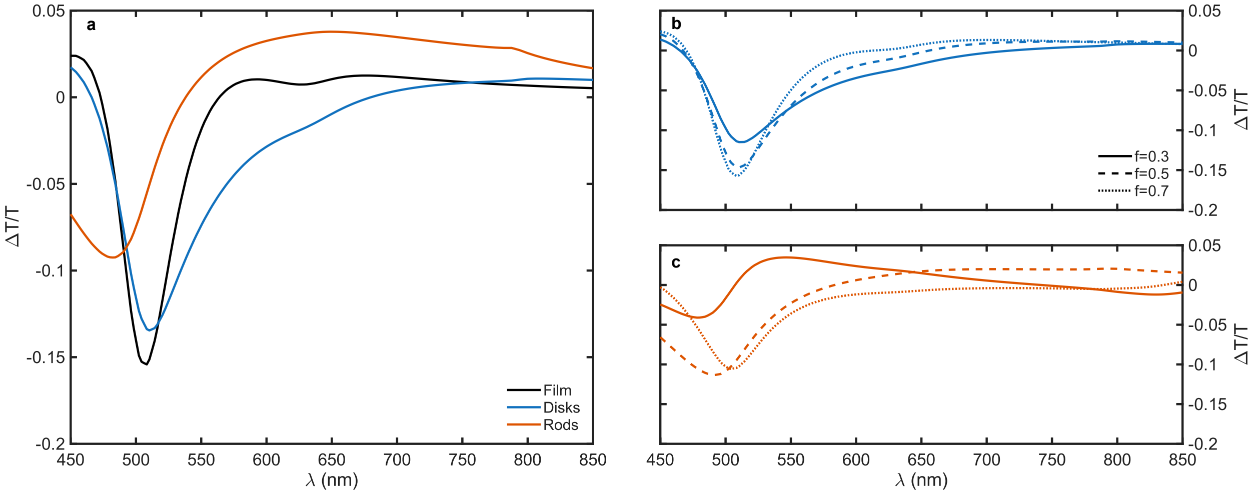

Supplementary Figure 9. **Generalization of the Bruggeman model. a** Calculated $\Delta T/T$ signal for the time delay corresponding to the maximum signal for each of three different systems: a BG film, disk-like inclusions and rod-like inclusions. Each system had a thickness of 30 nm, and the two latter a filling factor of 0.4. **b,c** The corresponding signal at different filling factors for the disks and the rods are also displayed, with filling factors as indicated in the figure.

Supplementary Note 8. Transient transmission as a function of filling factor of nanoporous gold

We calculated the $\Delta T/T$ signal for different metal filling factors using a thickness of 30 nm and a fluence of 3 mJ cm^{-2}. In Supplementary Figure 9, the calculated signal, taken at the time delay corresponding to its maximum modulation at each filling factor, is shown as a function of both wavelength and filling factor. In the figure, the zero-crossing is also highlighted. The figure illustrates how we can engineer properties of NPG, in this case the zero-crossing of the $\Delta T/T$ signal, by changing the filling factor of the film.

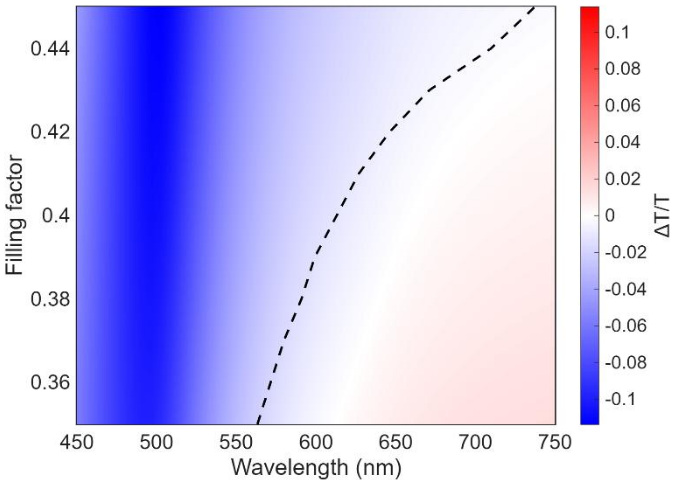

Supplementary Figure 10. **Calculated $\Delta T/T$ for different filling factors, determined at the time delay corresponding to the maximum modulation of the signal for each case.** The dashed line highlights the zero-crossing of the calculated signal.

Supplementary References

1. Moulder, J. F., Stickle, W. F., Sobol, Peter E., & Bomben, Kenneth D. *Handbook of X-Ray Photoelectron Spectroscopy: A Reference Book of Standard Spectra for Identification and Interpretation of XPS Data*. (Perkin-Elmer Corporation, Eden Prairie, Minn, 1995).
2. Rakic, A. D., Djurisic, A. B., Elazar, J. M. & Majewski, M. L. Optical properties of metallic films for vertical-cavity optoelectronic devices. *Appl. Opt.* **37**, 5271–5283 (1998).
3. Marini, A. *et al.* Ultrafast nonlinear dynamics of surface plasmon polaritons in gold nanowires due to the intrinsic nonlinearity of metals. *New J. Phys.* **15**, 013033 (2013).
4. Zavelani-Rossi, M. *et al.* Transient Optical Response of a Single Gold Nanoantenna: The Role of Plasmon Detuning. *ACS Photonics* **2**, 521–529 (2015).
5. Schirato, A. *et al.* Transient optical symmetry breaking for ultrafast broadband dichroism in plasmonic metasurfaces. *Nat. Photonics* **14**, 723–727 (2020).
6. Rosei, R. Temperature modulation of the optical transitions involving the Fermi surface in Ag: Theory. *Phys. Rev. B* **10**, 474–483 (1974).
7. Guerrisi, M., Rosei, R. & Winsemius, P. Splitting of the interband absorption edge in Au. *Phys. Rev. B* **12**, 557–563 (1975).
8. Dal Conte, S. *et al.* Disentangling electrons and lattice nonlinear optical response in metal-dielectric Bragg filters. *Phys. Rev. B* **89**, 125122 (2014).
9. Markel, V. A. Introduction to the Maxwell Garnett approximation: tutorial. *JOSA A* **33**, 1244–1256 (2016).
